# Evaluation of antibiotic activity of methicillin in healing of full-thickness infected wounds with sensitized methicillin resistant *Staphylococcus aureus* in the presence of HAMLET 

**DOI:** 10.22038/IJBMS.2018.27751.6764

**Published:** 2018-10

**Authors:** Amir Amniattalab, Rahim Mohammadi

**Affiliations:** 1 Young Researchers and Elite Club, Urmia Branch, Islamic Azad University, Urmia, Iran; 2 Department of Surgery and Diagnostic Imaging, Faculty of Veterinary Medicine, Urmia University, Urmia, Iran

**Keywords:** HAMLET, Infection, Methicillin resistant-S. *aureus*, Rat, Wound healing

## Abstract

**Objective(s):**

The novel healing choices for handling of infections due to multidrug resistant *Staphylococcus aureus* are reguired. HAMLET has been reported to be able to sensitize bacterial pathogens to traditional antimicrobial agents. The aim was to assess wound healing activity of methicillin in presence of HAMLET in methicillin resistant *S. aureus* (MRSA) infected wounds.

**Materials and Methods:**

Fifty male rats were randomized into five groups of ten animals each. In CONTROL group, 0.1 ml sterile saline 0.9% solution was added to the wounds with no infection. In MRSA group, the wounds were infected with MRSA and only treated with 0.1 ml the sterile saline (0.9%) solution. In MRSA/HAMLET group, infected wounds were cured with HAMLET (100 µg). In group MRSA/ Met, animals with infected wounds were cured with 0.1 ml local use of 1 mg/ml methicillin. In MRSA/Met/HAMLET group, animals with infected wounds were cured with local use of 0.1 ml solution of methicillin (1 mg/ml) and HAMLET (100 µg). All test formulations were used for ten consecutive days, twice a day, beginning from first treatment.

**Results:**

Microbiological examination, planimetric, histological and quantitative morphometric studies, immunohistochemical staining for angiogenesis, determination of hydroxyproline levels and RT-PCR for Caspase 3, Bcl-2 and p53 showed that there was significant difference between animals in MRSA/Met/ HAMLET group compared to other groups (*P*<0.05).

**Conclusion:**

HAMLET could make methicillin beneficial for handling of MRSA infected wounds and had the prospective effect to consider this harmless agent for local application.

## Introduction

The open wounds are susceptible to infection, especially by bacteria, that may result in systemic infections. Wounds that are infected by bacteria may be cured slowly and frequently may result in production of undesired exudates and toxins created by destruction of regenerating cells. 

Accelerated healing of the damaged wound and restoration of its normal function is desired (1). *Staphylococcus aureus* is reported to be an important source of nosocomial infections in hospitals (2).

It has been reported that Methicillin-resistant *S. aureus* (MRSA) is the most extensive bacterial pathogen affecting numerous infections in damaged skin (3, 4). Infections could result in sever mortality (5, 6). The pharmaceutical industry offers few new antibacterial agents (7). Most antibiotics produced in the recent years bear molecules that cannot overcome resistance mechanisms of the bacteria (8). Hence, operational novel curative alternatives for handling of *S. aureus* induced infections seem crucial.

Recently, it has been demonstrated that human alpha-lactalbumin made lethal to tumor cells (HAMLET) bears anti-bacterial properties *in vitro*. It has also been reported that HAMLET bears capacity of sensitization of bacterial pathogens that are resistant to conventional antibiotics *in vitro* (9). HAMLET along with present antibiotics has been effective against multi-drug-resistant staphylococci both *in vitro *and* in vivo* (10).

We planned this study to assess wound healing activity of methicillin in presence of HAMLET in MRSA infected wounds in rats and based on our literature review, this was the first *in vivo* study of this kind in the literature.

The assessments were based on excision wound model and planimetric studies, histomorphometric analyses, immunohistochemical staining for angiogenesis, determination of hydroxyproline levels and reverse transcription polymerase chain reaction (RT-PCR) for Caspase 3, Bcl-2 and p53.

## Materials and Methodsd

***Ethical considerations***

Our study was done based on recommendations in the Guide for the Care and Use of Laboratory Animals of the National Institutes of Health. Our study protocol was presented to the Institutional Committee and was admitted under license number of 95125 - 1396/30/9. All procedure was done under settings to diminish any probable distress and pain of the animals.

***Reagents and microorganisms***

All antibiotics and reagents were research grade and purchased from Sigma-Aldrich, St Louis, MO and employed without extra purification. The MRSA ATCC 43300 strain purchased from ATCC® 43300MINIPACK

™, Manassas, VA 20108 USA. The methicillin stock was prepared by diluting at least 100-fold in phosphate buffered saline (PBS), pH 7.4, prior to usage in the analyses.

***Purification of HAMLET***

HAMLET was purified according to others method (11). First, partially unfolded alpha-lactalbumin was treated with EDTA. This was performed in the presence of oleic acid (C18:1) on an anion-exchange matrix. The aim was to prepare a firm protein-lipid complex. Then it was resuspended in PBS for all experimentations. 

*The Escherichia coli *(BL21 DE3 pLysS) was used to purify a-lactalbumin. The *E. coli* was first induced with isopropyl β-D-1-thiogalactopyranoside (1 mM). The *E. coli *that we used was carrying vector pALA with the complete human alpha-lactalbumin gene inserted between the *Nde*I (site 100) and *Eco*RI (site 499) sites of the pAED4 vector. One liter of culture medium was utilized to isolate inclusion bodies. The inclusion bodies were dissolved in 40 ml of buffer (8M urea 10 mM TrisHCl 10 mM reduced glutathione, pH 8.0), and applied to a DEAE cellulose column. We eluted the protein and used 10 mM reduced glutathione in a dropwise manner to reduce the protein. 500 ml of folding buffer (10 mM TrisHCl 1 mM CaCl_2_ 100 mM KCl 10 mM reduced glutathioney1 mM oxidized glutathione 20% glycerol, pH 8.0, at room temperature) was consumed. Following entire folding of the protein, it was treated by 10 mM EDTA. The resulting suspension was used in a phenyl-Sepharose column and a-lactalbumin was eluted with 1 mM CaCl_2_.

***The procedures for wound creation and infection***

Animals, 4 weeks of age and approximately 180 g, were anesthetized by an intraperitoneal injection of ketamine (70 mg/kg of BW) and xylazine (5mg/kg of BW), and the hair of the place was callipered cleansed. After surgical prep, the skin was excised in circle and a wound with about 115 mm^2^ full thickness area was created on the anterior-dorsal side of each animal. Each wound was then inoculated with 5 × 10^7^ CFU of *S. aureus *ATCC 43300. We put a sterile gauze on the wound and the wound was stitched using 4-0 nylon sutures. This operation resulted in a local abscess after 24 hr. The animals were sent back to their cages and they were monitored on a daily basis. Following 24 hr, we opened the wounds and sent the gauze for quantitative bacterial cultures. We immediately started treatment of the wound.

***Animal grouping***

Fifty male rats were randomized into five groups of ten animals each. In CONTROL group, 0.1 ml the sterile saline 0.9% solution was added to the wounds with no infection. In MRSA group, the wounds were infected with methicillin resistant *S. aureus *ATCC 43300 and only treated with 0.1 ml the sterile saline 0.9% solution. In MRSA/HAMLET group, the infected wounds were treated with HAMLET (100 µg). In group MRSA/ Met, the animals with infected wounds were cured with 0.1 ml local use of 1 mg/ml methicillin. In MRSA/Met/HAMLET group, the animals with infected wounds were cured with local use of 0.1 ml solution of methicillin (1 mg/ml) and HAMLET (100 µg). All the ointments were used for ten consecutive days, twice a day, beginning from the first treatment. 

***Microbiological examination***

Briefly, for total bacterial count on days 7 and 14 of treatment after wound creation the granulated tissues were excised aseptically. Then, 0.1 g of sample was crushed and homogenized in sterile mortar containing 10 ml of sterile saline. The homogenized sample was serially diluted in tube containing 9 ml of sterile saline to 10-5. The diluted samples were cultured on plate count agar (Merck KGaA, Darmstadt, Germany) superficially and duplicated. We incubated the cultured plates at 37 ºC for one to two days. After incubation, all colonies were counted and results described as CFU/g of granulation tissue (12).

***Excision wound model and planimetric studies***

The percentage of wound contraction and clause time was analyzed with taking digital photos on from day 0 to day 21 every three days. We used a ruler near the wounds as a scale. The area of the wounds was measure by means of Measuring Tool of Adobe Acrobat 9 Pro Extended software (Adobe Systems Inc, San Jose, CA, USA) and the percentage of the contraction of the treated wounds were measured using the following equation: 

Wound contraction (%) = (A_0_ – A_t_ ) / A_0_ × 100 

Where A_0_ is the original wound area and A_t_ is the wound area at the time of imaging. 

***Histological preparation and quantitative morpho-metric studies***

The tissue samples were taken on three time points of 7, 14, 21 days after wound creation. The samples were stained with hematoxylin and eosin (H&E) and Masson’s trichrome. The morphometric indices including cellular infiltration and fibroblastic aggregation were quantitatively assessed. Qualitative parameters were classified based on the others including acute hemorrhage, congestion, vascularization, epithelialization, collagen production and density were also assessed using image analyzing software (Image-Pro Express, version 6.0.0.319, Media Cybernetics, Silver Springs, MD, USA) (13).

***Immunohistochemical staining for angiogenesis***

We heated the sample sections for 25 min at 60 ^°^C inside an oven with hot air. The samples were dewaxed and under gone alcohol gradient for rehydration. We used 10 mM sodium citrate buffer for antigen antigen retrieval. Based on the manufacturer’s instructions, the staining was performed. The blockage of the endogenous peroxidase was achieved using 0.03% hydrogen peroxide containing sodium azide for five min. We used a washing buffer to wash the samples. Then, samples were incubated with primary antibodies (CD31 biotinylated) and the incubation lasted for a quarter of hr. We used a washing buffer to wash the samples and put them in a buffer bath. We supplied a chamber with adequate load of streptavidin–HRP and placed the samples there. The incubation lasted for a quarter of hr. Again we used a washing buffer to wash the samples and put them in a buffer bath. We added a DAB chromogen to the samples and again incubation lasted for five min. We used a washing buffer to wash the samples and counter stained the samples with hematoxylin for five sec. We dipped the samples for 10 times in ammonia 0.037 M. Then the samples were rinsed with distilled water and cover slipped. The samples were observed under a light microscope.

***Determination of hydroxyproline amounts in tissue samples***

 We estimated the amounts of hydroxyproline according to works of others (13). For estimation of the hydroxyproline amounts UV-visible spectrophotometer (CamSpec M330, Cambridge CB2 4BG, UK) at 557 nm was used.

***RNA isolation and cDNA synthesis***

We extracted the total RNA from the samples. This process was performed according to a method known as standard TRIZOL. About 50 to 100 mg of the sample tissue was homogenized using 1 ml TRIZOL. We carefully collected the colorless aqueous phase in order not to contaminate DNA. A spectrophotometer was used to estimate content of RNA at 260 nm. The separated RNA was stored at - 70 ^°^C. In order to perform the RT-PCR, based on the manufacturer’s instructions, we synthesized cDNA in a 20 µl reaction mixture with 1 µg RNA, oligo (dT) primer (1 µl), 5×reaction buffer (4 µl), RNAse inhibitor (1 µl), 10 mM dNTP mix (2 µl) and M-MuLV Reverse Transcriptase (1 µl). The instruction for cycling for 20 µl reaction mix included five min at 65 ^°^C and then 60 min at 42 ^°^C and finally five min at 70 ^°^C.

***Reverse transcription polymerase chain reaction (RT-PCR) for caspase-3, Bcl-2 and p53***

We performed the PCR in a volume of 25 μl with PCR master mix (12.5 μl), reverse and forward specific primers, and cDNA as a template (1 μl) and nuclease free water (10 μl). The conditions were set as follow: Three min at 95 ^°^C for general denaturation, then 40 cycles of 95 ^°^C for 20 sec. The temperature for annealing was set at 62 ^°^C for Bcl-2, 52 ^°^C for *p53*and 50 ^°^C for caspase-3 that lasted for 60 sec. For elongation, the conditions were 72 ^°^C for 1 min and 72 ^°^C for 5 min. At the end, the products of the reaction were isolated using 1.5 % agarose gel. They were visualized using ethidium bromide staining with Gel Doc 2000. Table 1 shows forward and reverse primers caspase-3, Bcl-2 and p53.

***Statistical analysis***

We evaluated differences among groups by Kruskal–Wallis variance analysis. We compared days with Mann–Whitney U-test and for possible multiple comparisons we used Bonferroni test. We used SPSS 11.5 (SPSS Inc., Chicago, IL, USA) for statistical analysis. We considered *P*<0.05 as significant level. 

## Results

***Microbiological examination***

In animals of MRSA/Met/HAMLET group whose infected wounds were treated with both methicillin and HAMLET, showed a significant lower the number of *S. aureus* cultured in the wound tissues than in the infected wounds of MRSA/HAMLET and MRSA/Met groups (*P*<0.05).

None of the rats were expelled form study because of over dose of the anesthesia. The uninfected wounds treated with saline had no CFU/g of *S. aureus* count. Topical application of 0.1 ml solution of methicillin (1 mg/ml) and HAMLET (100 µg) significantly reduced the rate of total bacterial count on 7 and 14 days post-wounding compared to MRSA/HAMLET and MRSA/Met groups (*P*<0.05) (Table 2). 

**Figure 1:**
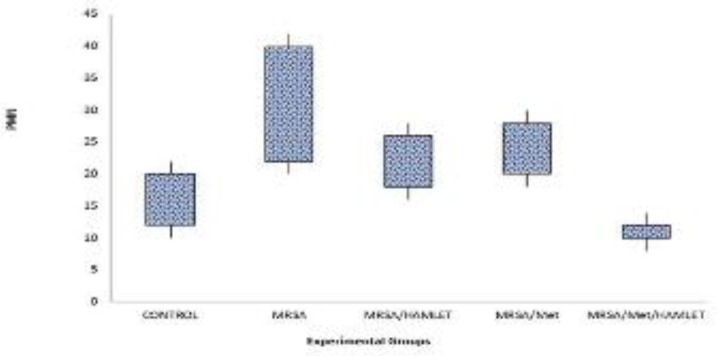
Box-and-whisker plots shows number of polymorphnuclear cells (PMN) in excisional model of the rat’s skin in experimental groups. Results were expressed as mean±SEM

**Figure 2:**
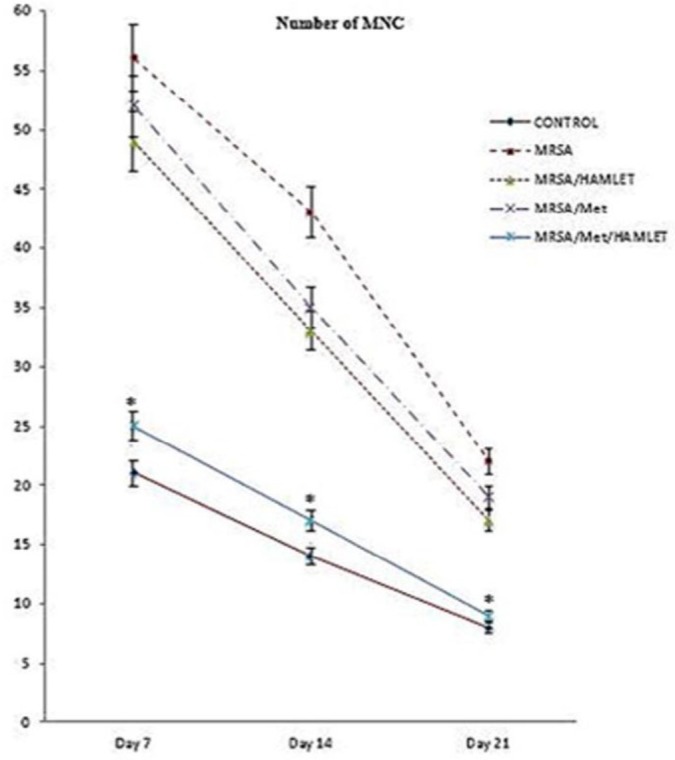
Line graph shows number of mononuclear cells (MNC) in excisional model of the skin of the animals in experimental groups. Results were expressed as mean±SEM. * *P*<0.05 vs MRSA/HAMLET and MRSA/Met groups

**Figure 3:**
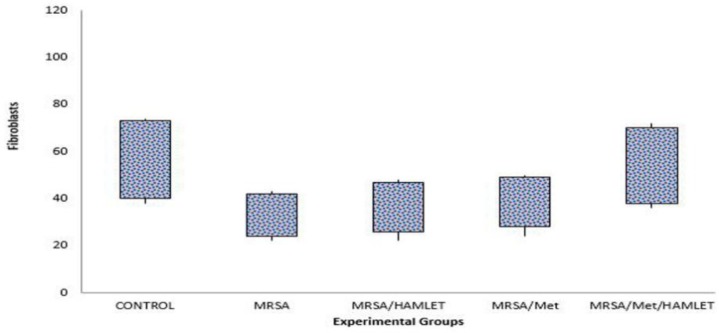
Box-and-whisker plots shows number of fibroblasts in excisional model of the rat’s skin in experimental groups. Results were expressed as mean±SEM

**Figure 4:**
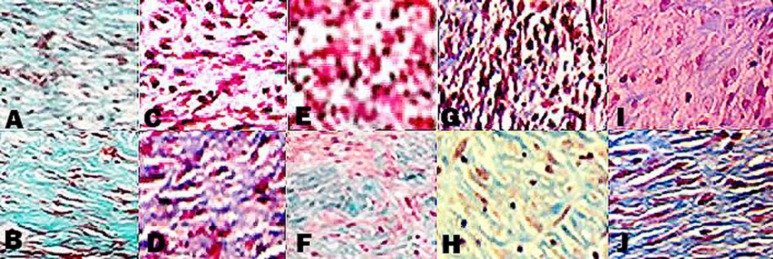
Histological properties of rat skin on the 7th (A,C,EG,I) and 14th day (B,D,F,H,J) after wound creation in excisional wound model. A and B: CONTROL, C and D: MRSA, E and F: MRSA/HAMLET, G and H: MRSA/Met, I and J: MRSA/Met/HAMLET. Masson trichrome staining (×400)

**Figure 5:**
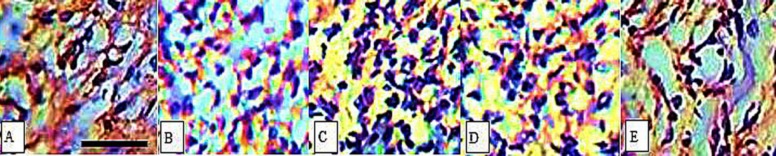
Immunohistochemical staining for CD31 on day 8 post wounding: (A) CONTROL, (B) MRSA, (C) MRSA/HAMLET, (D) MRSA/Met and (E) MRSA/Met/HAMLET. Note the elevated vascular distribution in MRSA/Met/HAMLET group. Scale bar: 50 µm

**Figure 6:**
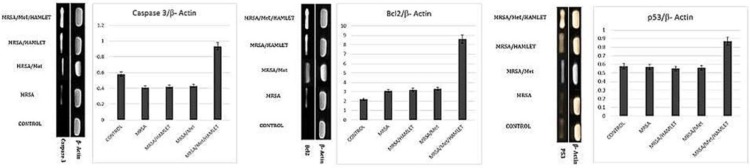
(Left) RT-PCR results for mRNA levels of caspase-3 based on β-Actin intensity. (Middle) RT-PCR results for mRNA levels of Bcl-2 based on β-Actin intensity. (Right) RT-PCR results for mRNA levels of p53 based on β-Actin intensity. Methicillin in presence of HAMLET enhanced the mRNA level of caspase 3, Bcl-2 and p53 on day 8 post-operation, all data are presented in Mean±SD. *P<0.05 vs MRSA/HAMLET and MRSA/Met groups

**Table 1:** Sequences of the primer pairs and product sizes used for RT-PCR

Gene name	Forward	Reverse	Product size
Caspase-3	5'-TACCCTGAAATGGGCTTGTGT-3'	5'-GTTAACACGAGTGAGGATGTG-3'	446 bp
Bcl-2	5' - CGCCCGCTGTGCACCGAGA-3'	5' -CACAATCCTCCCCCAGTTCACC-3'	228 bp
P53	5′-GAGGAGATGATGCTGCTGAG-3′	5′-TGCTGCTGCTGCTATTACC-3′	250 bp

**Table 2:** Wound bacterial count in experimental groups on days 7 and 14

**Groups**	**Wound bacterial count (CFU/g) of granulation tissue**	
	**Day 7**	**Day 14**
**CONTROL**	0.00 ± 0.00	0.00 ± 0.00
**MRSA**	1334.54 ± 216.73	1002.75 ± 237.84
**MRSA/HAMLET**	1077.39 ± 231.11	957.17 ± 289.15
**MRSA/Met**	1221.92 ± 241.19	978.29 ± 235.18
**MRSA/Met/HAMLET**	176.89 ± 42.19*	00.00 ± 0.00*

CFU: Colony-forming units.

* *P*<0.05 *vs*. MRSA/HAMLET and MRSA/Met groups.

**Table 3:** Effect of topical application of methicillin (1 mg/mL) and HAMLET (100 µg) on circular excision wound contraction area (mm^2^). Values are given as mean ± SEM

**Wound area in days (mm** ^2^ **)**						
**Groups**	**Day 6**	**Day 9**	**Day 12**	**Day 15**	**Day 18**	**Day 21**
**CONTROL**	257.56±4.98	105.35±5.18	88.72±3.68	42.72±3.35	24.28±2.15	7.88±3.36
**MRSA**	257.15±4.68	205.81±4.43	185.18±3.26	149.72±3.98	99.68±3.43	77.18±3.76
**MRSA/HAMLET**	225.21±4.18	193.76±4.77	174.85±3.26	123.57±2.18	71.68±2.17	62.39±2.84
**MRSA/Met**	227.25±4.15	195.55±4.57	177.38±3.19	127.55±3.68	73.12±2.15	64.62±2.27
**MRSA/Met/HAMLET**	115.19±3.15*	74.65±2.17*	31.77±2.27*	14.37±1.56*	4.53±0.78*	0.00±0.00*

The treated groups are compared by Student t test with other groups.

* : The mean difference is significant at the .05 level *vs*. MRSA/HAMLET and MRSA/Met groups.

**Table 4:** Intensity of histological parameters assessed in experimental animals

**Histological parameters**						
**Groups**	**Days**	**Acute Hemorrhage**	**Congestion**	**Vascularization**	**Epithelialization**	**Collagen**
**CONTROL**	7	+++	++++	+	-	+
	14	++	+	++	+	++
	21	-	-	+++	++	++
**MRSA**	7	++++	++++	-	-	-
	14	+++	+++	+	+	+
	21	++	++	+	+	+
**MRSAHAMLET**	7	++++	++++	-	-	-
	14	+++	++	+	+	+
	21	++	++	+	+	+
**MRSA/Met**	7	+++	+++	-	+	+
	14	++	+	+	++	++
	21	+	+	+	++	++
**MRSA/Met/HAMLET**	7	+*	+*	+++*	++*	++*
	14	-	-	++++*	+++*	+++*
	21	-	-	++++*	++++*	++++*

Classification of histological parameters according to the intensity of occurrence:

Histopatological damages were assessed as explained under material and methods on days, 7, 14 and 21 of lesion.

- absence;

+ discrete;

++ moderate;

+++ intense;

++++ very intense.

* *P*<0.05 *vs*. MRSA/HAMLET and MRSA/Met groups.

**Table 5:** Mean distribuion of vessels per one mm^2^ in the wound area on day 8 post-operation. All data are presented in Mean±SD

**Groups**	**Vessels**
CONTROL	9.00±1.25
MRSA	7.25±1.75
MRSA/HAMLET	8.25±1.50
MRSA/Met	7.75±1.50
MRSA/Met/HAMLET	19.25±1.75*

* *P*<0.05 *vs*. MRSA/HAMLET and MRSA/Met groups

***Diminishing of wound area***

Diminishing of wound are percentage in various groups within the study period and is displayed in Table 3. The rate of process of healing of wounds in MRSA/Met/HAMLET group was significantly different compared to MRSA/HAMLET and MRSA/Met groups (*P*<0.05).

***Histological and morphometric findings***

When MRSA/Met/HAMLET and MRSA/Met groups were compared regarding infiltration of cells, acute hemorrhage, congestion, edema, collagen production and density, reepithelialisation and neovascularization, a significant difference was observed (*P*<0.05). In the interval of study, the values for reepithelialisation and neovascularisation were significantly higher in MRSA/Met/HAMLET group than MRSA/HAMLET and MRSA/Met groups (*P*<0.05). 

In MRSA/Met/HAMLET group, the cellular count [polymorphonuclear (PMN) and mononuclear (MNC)], proliferation of fibroblasts and the qualitative study of acute hemorrhage, edema and collagen production values indicated a significant increase compared to those of MRSA/HAMLET and MRSA/Met groups (*P*<0.05) (Table 4) (Figure 1-4).

***Findings of immunohistochemical staining for angiogenesis***

Immunohistochemical analyses showed that topical application of 0.1 ml solution of methicillin (1 mg/ml) and HAMLET (100 µg) remarkably up-regulated the angiogenesis (*P*<0.05) (Table 5) (Figure 5).

***Hydroxyproline amounts in tissue samples***

Hydroxyproline amounts in the CONTROL, MRSA, MRSA/HAMLET, MRSA/Met, and MRSA/Met/HAMLET were found to be, respectively, 47.65±2.31, 63.47±2.82, 72.17±3.19, 70.17±2.16 and 99.78±3.36 mg per g. Hydroxyproline amounts were significantly augmented in the MRSA/Met/HAMLET group which denotes more collagen deposition in comparison with MRSA/HAMLET and MRSA/Met groups (*P*<0.05). 

***RT-PCR results for caspase-3, Bcl-2 and p53***

In order to evaluate the cell proliferation ratio on day 8 after wound creation, the mRNA levels of caspase-3, Bcl-2 and p53 genes were analyzed. Observations demonstrated that topical application of 0.1 ml solution of methicillin (5 mg/ml) and HAMLET (100 µg) resulted in a significant increase at caspase-3 mRNA level versus control group (*P*<0.05). The animals in MRSA/Met/HAMLET group showed a remarkable enhancement at mRNA level of Bcl-2 and p53 in comparison with than MRSA/HAMLET and MRSA/Met groups (*P*<0.05) (Figure 6). 

## Discussion

The findings of our investigation demonstrated that animals cured with HAMLET had inferior counts of *S. aureus* compared to others. The diminishing rate of wound area in animals cured with HAMLET was more than others. A significant difference was observed between MRSA/Met/HAMLET and MRSA/Met groups regarding the cellular count [polymorphonuclear (PMN) and mononuclear (MNC)], proliferation of fibroblasts and the qualitative study of acute hemorrhage, edema and collagen production values. Immunohistochemical observations indicated upregulation of angiogenesis in animals cured with HAMLET. Hydroxyproline amount of wound was markedly increased in HAMLET treated animals. HAMLET treatment resulted in a significant increase at caspase-3 mRNA level versus control animals.

The process of healing of wound could be explained by reepithelialization, growth of granulation tissue and restoration of extracellular matrix. The process of healing of wound take place spontaneously, and does not need much assistance, however, there are different risk factors like bacterial contamination, blood deprivation and malnutrition can impact the improvement of this trend (14). Microbial contaminations, could interrupt the natural process in healing of wound (15). MRSA is rising in infections and is a thoughtful danger to patients in hospitals and the public. Therefore, handling of MRSA infections seems to be expensive and problematic. The core result perceived in our study showed that contraction of the wounds cured with methicillin and HMLET was faster. Presence of necrotic tissue, clotting and crust, reepithelialization and granulation tissue growth and bacterial count were affected; signifying that methicillin in presence of HAMLET was effective against MRSA. Topical application of HAMLET in the wound resulted in noteworthy wound healing activity, signifying that it could have sensitized MRSA to methicillin.

We observed a noteworthy diminish in excisional wound model in terms of reduction in area of wound. This is signifying that maturation of collagen might have been occurred via augmented cross linking. The synthesis and breakdown balance and deposition of collagen is significant in the process of healing of wound and expansion of strength of wound (16). Hydroxyproline is a foremost constituent of the collagen that allows the sharp twisting of the collagen helix. It supports steadiness in the triple-helical structure of collagen via creating hydrogen bonds. Hydroxyproline amount has been used as gauge to estimate amount of collagen (17). Rise in hydroxyproline amount in MRSA/Met/HAMLET group designated augmented collagen amount, since hydroxyproline is the straight approximation of production of collagen.

Our preliminary data showed that methicillin in presence of HAMLET significantly reduced tissue bacteria count and promoted the healing stages. Accordingly, the animals in MRSA/Met/HAMLET group showed shortened homeostasis and inflammatory phases and accelerated proliferation and maturation stages. Considering the importance of the bacterial infection as well as presence of pathogens in wound tissue, we analyzed the MRSA colonies count in wound area. The observations demonstrated that the infection was controlled after administration of the methicillin combined with HAMLET. 

The inflammation phase is measured as a foremost stage to remove cellular debris from tissue and extensive response for microbial infection (18, 19). Therefore, rapid inflammatory response is necessary to control the inflammation. Neutrophils, macrophages and lymphocytes infiltrate to the site of injury during inflammatory stage (19, 20). Light microscopic analyses showed that in MRSA/Met/HAMLET group mononuclear immune cell infiltration was significantly increased on day 8 post operation. This situation plays a critical role in eliminating the infection and provoking the healing process by considering the key role of inflammatory cells (especially macrophages) in organizing the granulation tissue. Therefore, the antibacterial impact of methicillin in the presence of HAMLET may largely correlates with these agents. 

The observations of our study showed that methicillin in presence of HAMLET resulted in enhanced cellular proliferation. The fibroblasts and fibrocytes distribution in one mm^2^ of the wound site was significantly higher in comparison with other groups. Regarding the key role of fibroblasts and fibrocytes in synthesis of collagen, we could hypothesize that elevated collagen deposition in MRSA/Met/HAMLET group was attributed to high cellularity of fibroblasts and fibrocytes. Increased neovascularization on day 8 post wounding indicated that methicillin in presence of HAMLET could induce the process of healing through motivating infiltration of cells after 8 days.

Our histochemical findings for vascular distribution were in agreement with these findings. The animals in group MRSA/Met/HAMLET exhibited remarkably higher vascularization compared to MRSA/Met and MRSA/HAMLET groups. Increased neovascularization on day 8 post wounding demonstrated that methicillin in the presence of HAMLET could induce the process of healing through motivating infiltration of cells after 8 days. 

The termination of the inflammation is the apoptotic activity of immune cells. Apoptosis is known a crucial module of numerous processes including normal cell turnover, proper development and functioning of the immune system, hormone-dependent atrophy, embryonic development, and chemical-induced cell death (21). The mediators could induce the infiltration of activated immune cells into inflammation site to protect the tissue against the pathogen infection in the inflammation response. Apoptosis of the immune cells and the apoptotic cells are cleared by macrophages at the end of the inflammation. The clearance by macrophages of cells, apoptosis is a key point phenomenon associated with actively tissue formation from wound inflammation (22).

The Bcl-2 family of proteins prohibits apoptosis as effectors of the apoptosis pathway (23, 24). In contrast, caspase and p53 that are the guardian of the genome, controls the fate of injured cells by spotting and stopping the cell cycle. Upon injury, the p53 and caspase are increased and induce apoptosis of the immune cells which in turn result in eliminating the immune cells. After this stage the Bcl-2 prohibits the apoptosis that triggers the cellular proliferation (18). Our RT-PCR analyses showed that in MRSA/Met/HAMLET group caspase 3, Bcl-2 and p43 expressions were increased. Thus, it could be concluded that methicillin in presence of HAMLET could enhance the cellular proliferation by up-regulating the caspase 3, Bcl-2 and p43 expressions. 

There are several reports that the HAMLET has sensitized the bacterial pathogens to traditional antimicrobial agents (9, 10, 25-27). However, all of these reports were performed in *in vitro* conditions and the literature lacks studies including sensitization of MRSA with HAMLET in *in vitro* set up. 

## Conclusion

The object of our investigation was to validate that methicillin in the presence of HAMLET could show antimicrobial activity against MRSA. This capability to rise the effectiveness of methicillin to the degree that drug-resistant *S. aureus* could again become sensitive to this antibiotic in *in vivo* assays is reported for the first time in the literature. Therefore, our findings showed that HAMLET could make methicillin beneficial for handling of MRSA infected wounds and had the prospective effect to consider this harmless agent for local application. Dose-response studies are needed to study various concentrations for the methicillin and HAMLET for determination of optimum dosages to achieve maximum effects.
